# The para-rectus approach for extraction of intra-pelvic migrated acetabular implants

**DOI:** 10.1093/jscr/rjaf091

**Published:** 2025-03-28

**Authors:** Mohamed Amine Selmene, Youssef Jaballah, Sabri Mahjoub, Hedi Annabi, Mourad Zaraa

**Affiliations:** Orthopaedic Department, Trauma and Burns Center, May 1^st^ Street, 2013, Ben Arous, Tunisia; Faculty of Medicine of Tunis, University Tunis El Manar, 15, Djebel Lakhdhar Street, Bab Saadoun, 1007, Tunis, Tunisia; Orthopaedic Department, Trauma and Burns Center, May 1^st^ Street, 2013, Ben Arous, Tunisia; Faculty of Medicine of Tunis, University Tunis El Manar, 15, Djebel Lakhdhar Street, Bab Saadoun, 1007, Tunis, Tunisia; Orthopaedic Department, Trauma and Burns Center, May 1^st^ Street, 2013, Ben Arous, Tunisia; Faculty of Medicine of Tunis, University Tunis El Manar, 15, Djebel Lakhdhar Street, Bab Saadoun, 1007, Tunis, Tunisia; Orthopaedic Department, Trauma and Burns Center, May 1^st^ Street, 2013, Ben Arous, Tunisia; Faculty of Medicine of Tunis, University Tunis El Manar, 15, Djebel Lakhdhar Street, Bab Saadoun, 1007, Tunis, Tunisia; Orthopaedic Department, Trauma and Burns Center, May 1^st^ Street, 2013, Ben Arous, Tunisia; Faculty of Medicine of Tunis, University Tunis El Manar, 15, Djebel Lakhdhar Street, Bab Saadoun, 1007, Tunis, Tunisia

**Keywords:** hip, acetabular components, intra-pelvic migration, para-rectus approach

## Abstract

Primary and revision total hip arthroplasty can be complicated by intrapelvic migration of the acetabular components. This complication constitutes an evolutionary turning point in the history of this prosthesis since it could lead to compression or invasion of noble intra-pelvic structures. The second problem lies in the extraction of these implants in this anatomically dangerous region. We report the case of a patient who was operated on for a septic loosening of her total hip arthroplasty with protrusion of the acetabular components (Kerboull cross-plate and the cup) intrapelvicly via the para-rectus approach which allowed safe removal of these implants. This is an intra-pelvis approach lateral to the rectus abdominis muscle, initially described in acetabular fractures, with few studies reporting its use in this type of complex situations.

## Introduction

Intrapelvic migration of acetabular implants is a rare and serious complication of prosthetic hip surgery [1]. It presents a therapeutic challenge relevant to extracting these implants from an anatomically dangerous region and the possible concomitant or subsequent acetabular reconstruction. Intra-pelvic approaches would be interesting ways of extracting these implants. Among them, the para-rectus approach, initially described in acetabular fractures surgery, constitutes an interesting alternative, with few studies in the literature reporting its interest in this subject [2].

We report the case of a patient operated on via the para-rectus approach to remove intra-pelvic migrated acetabular prosthetic implants.

## Case presentation

**Figure 1:**
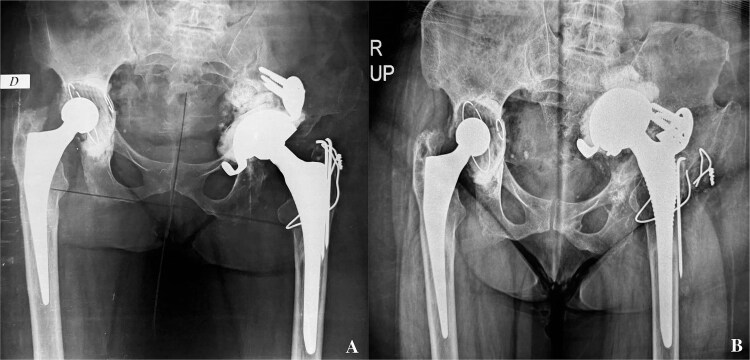
A- revision of the left total hip arthroplasty: postoperative pelvis X-ray. B- pelvis X-ray: Intrapelvic migration of acetabular components (Kerboul cross-plate and the cup).

This was a 49-year-old female patient, followed for ankylosing spondylitis, with a history of bilateral total hip arthroplasty. The right side was revised at 13 years post-operative for aseptic loosening of the femoral stem, and the left side was revised at 15 years post-operative for aseptic loosening of the acetabular component. During the latter operation, an acetabular reconstruction with a fragmented bone graft was performed associated to a Kerboull cross-plate (Fig. 1).

At 4 years post-operative, the patient consulted with left hip pain, walking discomfort and a return to the use of crutches for the past 6 months. On clinical examination, the scar was clean and solid. A lameness of both dodging and discrepancy of lower limbs was observed. Left hip mobility was reduced. Standard radiographs of the pelvis showed intrapelvic migration of a broken Kerboull cross-plate and the acetabular cup. The femoral stem was also loose (Fig. 1). The C-reactive protein was 68 and leukocytes were 11 200. Pelvic angio-CT-scan showed multiple periprosthetic collections. The whole formed a 13x8 cm magma repressing the bladder, sigmoid and uterus and the lateral iliac vessels.

**Figure 2:**
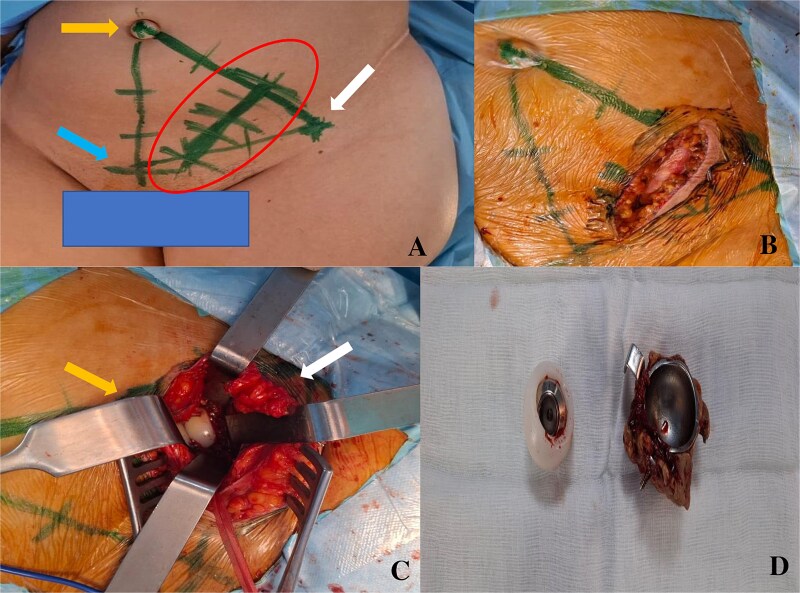
A- drawing of the scar of the para-rectus approach. Bony landmarks: right arrow: anterior superior iliac spine, up left arrow: umbilicus, bottom left arrow: pubic symphysis. B- scar size. C- intraoperative view of the acetabular implant before their extraction. The left arrow corresponds to the medial part where there are the lateral iliac vessels protected. The right arrow corresponds to the lateral part with the iliopsoas muscle spread apart. D- acetabular implants removed.

The para-rectus approach was performed to remove the acetabular components. Implants were highlighted from the second window of this approach where the lateral iliac vessels were protected medially and the iliopsoas muscle were spreaded laterally. The Kerboull cross-plate with cement bonded to it and the cup were safely removed, after ensuring that they were completely free of any attachment (Fig. 2). Bacteriological samples and thorough surgical washing were performed. The femoral stem was removed via the posterolateral hip approach. The germ identified was *Staphylococcus aureus* Meti-S, for which the patient received appropriate antibiotics. At 6 months’ follow-up, she was walking with a walker, with clinical and biological improvement, and was awaiting revision surgery.

## Discussion

Intrapelvic migration of acetabular implants is rare but could cause serious complications relevant to compression or invasion of endopelvic structures [1]. A meticulous preoperative preparation based on a good clinical, biological and imaging examination in necessary. Angio-CT-scan of the pelvis is a key examination in this context, enabling the detection of vascular abnormalities such as false aneurysms or arteriovenous fistulas in contact with the acetabular support ring or cup, cement pieces or screws. It can also be used to assess relationships with nerve, genitourinary and digestive structures [1].

The choice of the approach to extract these implants safely is crucial. Conventional hip approaches are indicated in case of moderate protrusion with no proximity with vital structures. In situations threatening noble structures, a specific approach is preferred [1]. According to Grigoris *et al.*, fortunately this migration is a slow process. So, a layer of fibrous tissue is formed between the implants, the vessels and the pelvic organs [3].

More than half of these cases are accompanied by prosthesis infection [1, 4]. Stiehl *et al.*, in their review of the literature, noted 11 cases of infection out of 16 severe protrusions of prosthetic acetabular implants [4].

In terms of specific approaches, Stiehl et al. has used the ilioinguinal and the Mears tri-radiated approach to remove acetabular components [5]. Grigoris *et al.* in 1997 described a retroperitoneal approach performed from the iliac crest with progression on the medial table of the iliac bone with retraction of the iliopsoas muscle medially. This approach requires conversion to intrapelvic in case of uncontrolled intraoperative bleeding [3]. Sarasin *et al.* removed an endopelvic acetabular cup via a laparotomy and trans-peritoneal approach with the help of a general surgeon [6].

More recently, the modified Stoppa midline intrapelvic approach has been used by Chana-Rodriguez *et al.* and Murcia-Asensio *et al.* with good control of pelvic structures and the possibility of reconstructing the acetabular defect [7, 8].

Keel *et al.* described the para-rectus approach as an intra-pelvic one, lateral to the rectus abdominis muscle, primarily to reduce and fix acetabular fractures [2]. In this type of pathology, this approach would be accompanied by a less blood loss, a shorter operative time and a smaller skin scar compared to the ilioinguinal approach and a shorter hospital stay with no difference in postoperative complications compared to the modified Stoppa approach [9, 10]. This approach exposes the anterior acetabular wall and the quadrilateral plate with a tangential view of the acetabulum [2]. This corresponds to the usual location of endopelvic protrusive implants.

To our knowledge, only one case reported the use of the para-rectus approach in the extraction of a migrated acetabular cup following aseptic loosening associated to a pelvic discontinuity [11]. This approach enabled these authors to perform extraction and acetabular reconstruction using a plate and support ring without postoperative complications. This was the case in our patient, but reconstruction of the acetabular defect was postponed until a later stage due to the infectious history.

In the literature, the indications for this approach have been extended to pelvic ring fractures, with particularly the sacroiliac involvement, exploration of lumbosacral nerve lesions and tumor pathology [12–15].

However, this approach has some limitations. Given the proximity of the peritoneum and intraperitoneal organs, there is a non-negligible risk of peritoneal perforation, making this surgery difficult in obese patients or those with abdominal distension, ileus, or bowel obstruction [2].

## Conclusion

Intrapelvic protrusion of acetabular components combines the risk of femur fracture during their extraction by conventional hip approach at the time of dislocation with the risk of injury to pelvic structures. These situations require the use of an abdominal approach for safe extraction. The intra-pelvic para-rectus approach is an interesting, safe and prudent alternative in this kind of complex situations.

## Data Availability

The data used to support the findings of this study are included within the article.
